# Quantitative comparison and reproducibility of pathologist scoring and digital image analysis of estrogen receptor β2 immunohistochemistry in prostate cancer

**DOI:** 10.1186/s13000-016-0511-5

**Published:** 2016-07-11

**Authors:** Anthony E. Rizzardi, Xiaotun Zhang, Rachel Isaksson Vogel, Suzanne Kolb, Milan S. Geybels, Yuet-Kin Leung, Jonathan C. Henriksen, Shuk-Mei Ho, Julianna Kwak, Janet L. Stanford, Stephen C. Schmechel

**Affiliations:** Department of Pathology, University of Washington, 908 Jefferson Street, Room 2NJB244, Seattle, WA 98104 USA; Department of Urology, University of Washington, Seattle, WA USA; Biostatistics and Bioinformatics Core, Masonic Cancer Center, University of Minnesota, Minneapolis, MN USA; Division of Public Health Sciences, Fred Hutchinson Cancer Research Center, Seattle, WA USA; Divison of Environmental Genetics and Molecular Toxicology, University of Cincinnati, Cincinnati, OH USA; Center for Environmental Genetics, Cincinnati Cancer Institute, University of Cincinnati, Cincinnati, OH USA; Department of Environmental Health, Cincinnati Cancer Institute, University of Cincinnati, Cincinnati, OH USA; Department of Epidemiology, School of Public Health, University of Washington, Seattle, WA USA; Department of Pathology, University of Washington, 300 Ninth Ave, Research & Training Building, Room 421, Seattle, WA 98104 USA

**Keywords:** Prostate cancer, Biomarkers, Digital pathology, Quantification, Estrogen receptor β2

## Abstract

**Background:**

Digital image analysis offers advantages over traditional pathologist visual scoring of immunohistochemistry, although few studies examining the correlation and reproducibility of these methods have been performed in prostate cancer. We evaluated the correlation between digital image analysis (continuous variable data) and pathologist visual scoring (quasi-continuous variable data), reproducibility of each method, and association of digital image analysis methods with outcomes using prostate cancer tissue microarrays (TMAs) stained for estrogen receptor-β2 (ERβ2).

**Methods:**

Prostate cancer TMAs were digitized and evaluated by pathologist visual scoring versus digital image analysis for ERβ2 staining within tumor epithelium. Two independent analysis runs were performed to evaluate reproducibility. Image analysis data were evaluated for associations with recurrence-free survival and disease specific survival following radical prostatectomy.

**Results:**

We observed weak/moderate Spearman correlation between digital image analysis and pathologist visual scores of tumor nuclei (Analysis Run A: 0.42, Analysis Run B: 0.41), and moderate/strong correlation between digital image analysis and pathologist visual scores of tumor cytoplasm (Analysis Run A: 0.70, Analysis Run B: 0.69). For the reproducibility analysis, there was high Spearman correlation between pathologist visual scores generated for individual TMA spots across Analysis Runs A and B (Nuclei: 0.84, Cytoplasm: 0.83), and very high correlation between digital image analysis for individual TMA spots across Analysis Runs A and B (Nuclei: 0.99, Cytoplasm: 0.99). Further, ERβ2 staining was significantly associated with increased risk of prostate cancer-specific mortality (PCSM) when quantified by cytoplasmic digital image analysis (HR 2.16, 95 % CI 1.02–4.57, *p* = 0.045), nuclear image analysis (HR 2.67, 95 % CI 1.20–5.96, *p* = 0.016), and total malignant epithelial area analysis (HR 5.10, 95 % CI 1.70–15.34, *p* = 0.004). After adjusting for clinicopathologic factors, only total malignant epithelial area ERβ2 staining was significantly associated with PCSM (HR 4.08, 95 % CI 1.37–12.15, *p* = 0.012).

**Conclusions:**

Digital methods of immunohistochemical quantification are more reproducible than pathologist visual scoring in prostate cancer, suggesting that digital methods are preferable and especially warranted for studies involving large sample sizes.

**Electronic supplementary material:**

The online version of this article (doi:10.1186/s13000-016-0511-5) contains supplementary material, which is available to authorized users.

## Background

Significant advances in digital imaging have enabled automated technologies to reproduce and often outperform pathologist visual scoring of immunohistochemistry (IHC) assays. Visual scoring has been the traditional gold standard method for quantifying IHC staining, but problems with this method include the limited range of resulting data [1, 2], human error [3], less than optimal reproducibility [4], and resulting ordinal or quasi-continuous variable data rather than true continuous variable data. Digital image analysis overcomes many of these limitations. For example, digital methods allow algorithm parameters to be locked yielding more reproducible data especially when staining is weak and most linearly related to antigen concentration [2, 5, 6], and output continuous variable data. Previous studies reveal that IHC cut-points of biomarkers with prognostic relevance may be identified using continuous variable digital imaging data that were either undetected [7] or not as strongly associated [2, 8–10] using visual scoring data. Furthermore, digital methods offer a feasible way to scale experiments to high-throughput sample sizes (e.g., experiments using tissue microarrays) which can be otherwise time-limiting for pathologists to complete [11].

Numerous studies have demonstrated a high degree of correlation between digital image analysis and pathologist visual scoring. The majority of this research has been performed in breast cancer tissue on human epidermal growth factor receptor, estrogen receptor, and progesterone receptor [8, 12–22]. Similar strong correlations between software algorithms and pathologist visual scoring have been described in other tissue types including esophageal cancer [23], colorectal cancer [24], ovarian cancer [11], and prostate cancer (PCa) [25].

Pathologist visual scoring data often use a simple ordinal variable scale (e.g., negative “0”, weak “1 + ”, medium “2 + ”, and strong “3 + ” positive staining). More complex pathologist visual scoring systems have been developed to provide quasi-continuous variable data, such as multiplying an ordinal variable of intensity by an estimate of tissue area comprising that intensity level [26, 27]. Although studies examining the correlation and reproducibility of pathologist visual scoring and digital image analysis have been performed in breast cancer, to date there has been little research validating such tools in PCa. Few prognostic biomarkers are available for routine clinical use in PCa and the use of digital methods for evaluating IHC assays in large PCa studies represents a valuable technique for evaluating protein biomarkers of tumor aggressiveness [28]. Estrogen receptor β2 (ERβ2) plays a metastasis-promoting role in PCa and has been demonstrated to have prognostic value for tumor progression [29, 30]. Here, we have evaluated the correlation between digital image analysis and pathologist visual scoring (using a semi-quantitative scoring technique [27]), the reproducibility of these two methods, as well as the association of digital image analysis with disease-specific survival using a large set of PCa tissue microarray (TMA) slides stained for ERβ2.

## Methods

### Clinical cohort and TMA construction

PCa patients (ages 35–74 at diagnosis) of European or African ancestry residing in King County, WA were identified from the Seattle-Puget Sound Surveillance, Epidemiology, and End Results (SEER) cancer registry for population-based studies of PCa risk factors after approval from the Fred Hutchinson Cancer Research Center Institutional Review Board [30–32]. All men were diagnosed with histologically confirmed PCa during either 1993–1996 or 2002–2005. Demographic information and medical history for each patient were obtained by structured in-person interviews, and clinicopathologic data were obtained from the cancer registry. Of the 831 interviewed patients who underwent radical prostatectomy, 566 (68 %) consented to release of tumor tissue including for TMA construction and had formalin-fixed paraffin-embedded blocks available for inclusion in this study, which maintains active Fred Hutchinson Cancer Research Center Institutional Review Board approval (IRF #4714) at the time of submitting this paper for publication. Vital status and underlying cause of death was available for these patients through the biannual linkages with the SEER registry and review of death certificates to confirm cancer-specific vs. other cause mortality. PCa recurrence was determined by follow-up surveys sent to patients in 2004–2005 and in 2010–2011, review of medical records, and physician follow-up as needed. Biochemical recurrence was defined as postoperative prostate-specific antigen (PSA) of ≥2.0 ng/mL. Metastatic progression was confirmed by bone scan, magnetic resonance imaging, computerized tomography scan, or biopsy.

Formalin-fixed, paraffin-embedded blocks of tumor tissue obtained at the time of radical prostatectomy were used to make hematoxylin and eosin (H&E) stained slides, which were reviewed by an experienced genitourinary pathologist. Duplicate tumor tissue cores of 1.0 mm diameter were taken from a single tumor focus (≥75 % tumor tissue) of the donor blocks and arrayed into a new recipient paraffin block with a manual tissue arrayer (MTA-1; Beecher Instruments, Sun Prairie, WI).

### Immunohistochemistry and pathologist visual scoring

Unstained 5 μm-thick TMA sections were deparaffinized and rehydrated using standard methods. IHC was performed on TMA sections using a custom polyclonal antibody specific for the C-terminus of ERβ2 (482-MKMETLLPEATMEQ-495) as previously reported [29]. ERβ2 stained slides were scanned for pathologist visual scoring using an automated Tissuefax microscope (TissueGnostics, Tarzana, CA) and reviewed via an online web gallery. ERβ2 immunostaining within malignant cells was scored for each TMA spot by a pathologist (X.Z.) blinded to clinical parameters. Cytoplasm and nuclei were evaluated separately. As described previously, immunostaining was assessed using a score calculated by multiplying staining intensity (0 for no staining, 1 for light/weak staining, and 2 for strong/intense staining) by the corresponding percentage of cells staining positive at each intensity (totaling to 100 %) [27]. Tissue spots that were missing, damaged, contained staining artifacts, or had uncertain histology were excluded from the analysis. Raw data for pathologist visual scores are included in Additional file 1.

### Slide digitization, annotation, and immunohistochemical quantification

For digital image analysis, TMA whole slide images were obtained at 40x magnification (0.0625 μm^2^/pixel) with a ScanScope CS (Aperio ePathology, Leica Biosystems Imaging, Vista, CA) and Genie Histology Pattern Recognition software (Aperio) was trained to classify tissues into Image Classes (tumor, stroma, and glass) as previously described [11]. ERβ2 staining in total malignant epithelial areas was quantified using the Color Deconvolution algorithm (Aperio) as the product of staining intensity (average optical density [OD] units) multiplied by the percentage of tumor epithelium with positive staining (denoted as AvgOD*%Pos). Cytoplasmic staining of ERβ2 within tumor epithelium was quantified using the Cytoplasmic algorithm (Aperio) as the product of staining intensity multiplied by the percentage of tumor epithelium with positive cytoplasmic staining (denoted as AvgCytoOD*%PosCyto). Similarly, nuclear staining was summarized as the average staining intensity within nuclei of tumor epithelium multiplied by the percentage of positive nuclei in tumor epithelium (denoted as AvgNuclearOD*%PosNuclei). These metrics have been previously described [11, 28, 33]. The amount of staining present is linearly related to OD [34].

### Reproducibility study

A blinded reproducibility study (Analysis Run B) was performed by the same pathologist (X.Z.) who rescored the TMAs using the above protocol (time period of 24 months between initial and repeat scoring). Similarly, the scientist who originally quantified the TMAs re-annotated the TMA spots, retrained the Genie Histology Pattern Recognition software, and reanalyzed the TMAs using the Cytoplasmic and Nuclear algorithms (time period of 10 months between initial and repeat scoring). Raw data for the digital image analyses are included in Additional file 1.

### Statistical analysis

ERβ2 IHC staining was evaluated by digital image analysis and pathologist visual scoring for comparison of quantification methods. The average score across duplicate spots was calculated for each case within each of the two Analysis Runs A and B. The association between pathologist scores and digital measures (AvgCytoOD*%PosCyto or AvgNuclearOD*%PosNuclei) was determined within and across Analysis Runs (A and B) using Spearman’s correlation coefficients, and point estimates and 95 % confidence intervals are presented. *P*-values represent a test of whether the correlation coefficients are statistically significantly different than 0 (no correlation). Associations of ERβ2 (quantified by image analysis confined to tumor cytoplasm, tumor nuclei, or total malignant epithelial areas) and PCa outcomes (recurrence-free survival [RFS] and prostate cancer-specific mortality [PCSM]) were evaluated using Kaplan-Meier analysis and the log-rank test. Image analysis methods were evaluated using Cox regression models adjusted for age at diagnosis (continuous), Gleason score (≤6, 7[3 + 4], 7[4 + 3], and ≥8), pathologic stage (local: pT2, N0/NX, M0; regional: pT3/pT4 or N1-3, M0), and diagnostic preoperative PSA level. Hazard ratios (HRs) and 95 % confidence intervals (CIs) were reported. A two-tailed *p*-value of <0.05 was considered statistically significant.

## Results

### Immunohistochemical staining

**Fig. 1:**
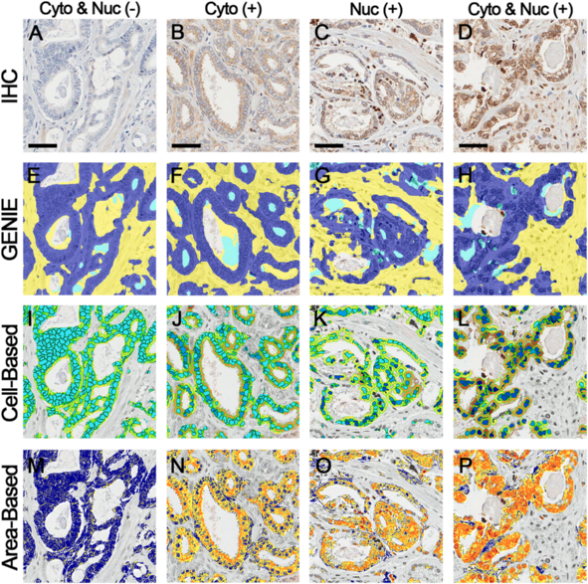
Image analysis workflow for immunohistochemical staining quantification. **a**-**d** Prostate cancer tissue microarrays were stained by immunohistochemistry (IHC). Various staining qualities are highlighted. **e**-**h** Genie Histology Pattern Recognition software (Aperio) subclassified tumor areas into malignant epithelium (dark blue), stroma (yellow), and glass (cyan). **i**-**l** Within malignant epithelial areas, cell-based digital image analysis separately quantified cytoplasmic and nuclear staining within malignant epithelium using the Cytoplasmic algorithm (Aperio). Cytoplasmic staining intensities are pseudocolored for negative cytoplasmic (yellow), weak cytoplasmic (orange), medium cytoplasmic (dark orange), and strong cytoplasmic (red) staining. Nuclear staining intensities are pseudocolored for negative nuclear (cyan), weak nuclear (light blue), medium nuclear (blue), and strong nuclear (dark blue) staining. **m**-**p** Within malignant epithelial areas, area-based digital image analysis quantified total malignant epithelial area staining using the Color Deconvolution algorithm (Aperio). Area-based staining intensities are pseudocolored for negative (blue), weak (yellow), medium (orange), and strong (red) staining. Scale bars represent 50 μm

ERβ2 was evaluated by IHC on the PCa patient cohort TMAs. In PCa tissue, ERβ2 displayed variable nuclear staining and variable finely granular cytoplasmic staining, both in malignant epithelial cells and in fibromuscular stromal cells (Fig. 1). In normal prostate tissue, ERβ2 displayed cytoplasmic staining in basal and luminal epithelial cells, agreeing with previously reported specificity and localization [29].

### Correlation of digital image analysis and pathologist visual scoring

Figure 1 demonstrates the workflow for digital image analysis (annotation, automated tissue classification, and image analysis). The digital image analysis process required approximately 1 min of a technician’s time for analysis of each TMA spot (under pathologist supervision) while the visual scoring process required approximately 1 min of a pathologist’s time for analysis of each TMA spot.

**Fig. 2:**
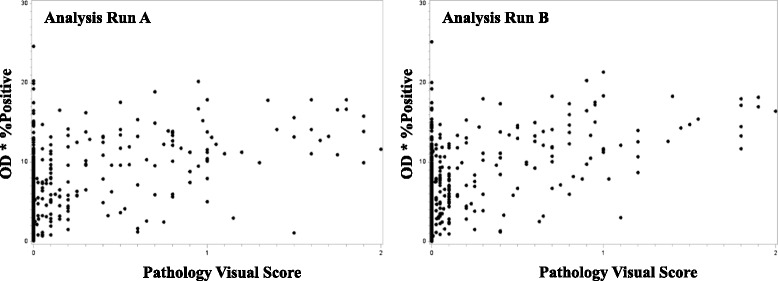
Correlation between digital image analysis and pathologist visual scoring of tumor nuclei. Scatter plots of nuclear data generated using digital image analysis (AvgNuclearOD*%PosNuclei) versus pathologist visual scores. Data were averaged across tissue microarray replicates for each patient for Analysis Run A (left) and Analysis Run B (right)

**Fig. 3:**
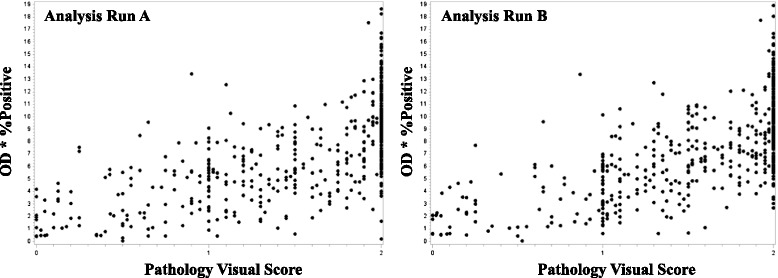
Correlation between digital image analysis and pathologist visual scoring of tumor cytoplasm. Scatter plots of cytoplasmic data generated using digital image analysis (AvgCytoOD*%PosCyto) versus pathologist visual scores. Data were averaged across tissue microarray replicates for each patient for Analysis Run A (left) and Analysis Run B (right)

Patients were represented in duplicate on the TMAs. Therefore we first compared the correlation of patient replicates to each other (within an Analysis Run). As shown in Table 1, the correlations between replicates were similar for both Analysis Runs A and B, and were higher for digital IHC measures (0.84 and 0.84 in tumor cytoplasmic areas in Analysis Runs A and B, respectively; and 0.85 and 0.84 in tumor nuclear areas in Analysis Runs A and B, respectively) compared to pathologist scores (0.72 and 0.71 in tumor cytoplasmic areas in Analysis Runs A and B, respectively; and 0.64 and 0.62 in tumor nuclear areas in Analysis Runs A and B, respectively). Both methods had relatively high correlation, indicating that replicates within a patient were similar. Since IHC evaluation is often performed for linking to outcome data on a per-patient level, these high correlations provided a rationale for averaging patient data together when comparing quantification methods. When quantification methods were directly compared (patient replicates averaged together), there was a weak/moderate correlation between digital IHC measures and pathology scores of tumor nuclei (Analysis Run A: 0.42 (0.34–0.49), *p* < 0.0001; and Analysis Run B: 0.41 (0.34–0.48), *p* < 0.0001; Fig. 2), and a moderate/strong correlation between digital IHC measures and pathology scores of tumor cytoplasm (Analysis Run A: 0.70 (0.65–0.74), *p* < 0.0001; and Analysis Run B: 0.69 (0.64–0.74), *p* < 0.0001; Fig. 3).

**Table 1:** Spearman correlation and 95 % confidence interval between two TMA replicates for each patient by Analysis Run

	Correlation (95 % CI)	
Digital IHC OD*%Pos	Analysis Run A	Analysis Run B
Cytoplasm	0.84 (0.81–0.86)	0.84 (0.81–0.86)
Nuclei	0.85 (0.82–0.87)	0.84 (0.81–0.87)
Pathologist Visual Score		
Cytoplasm	0.72 (0.68–0.76)	0.71 (0.66–0.75)
Nuclei	0.64 (0.59–0.69)	0.62 (0.57–0.68)

### Reproducibility of quantification methods

To assess the reproducibility of these methods, we performed a second independent analysis of the ERβ2 stained TMAs (Analysis Run B). Comparing data between Analysis Runs A and B, there was a high correlation between pathologist visual scores generated for individual TMA spots (0.84 for tumor cytoplasmic areas and 0.83 for tumor nuclear areas for Analysis Runs A and B, respectively), and very high correlation between digital IHC measures generated for individual TMA spots (0.99 for tumor cytoplasmic areas and 0.99 for tumor nuclear areas for Analysis Runs A and B, respectively) as shown in Table 2.

**Table 2:** Spearman correlation and 95 % confidence interval between Analysis Run A and B for the same TMA spot

Digital IHC OD*%Pos	Correlation (95 % CI)
Cytoplasm	0.99 (0.986–0.990)
Nuclei	0.99 (0.992–0.995)
Pathologist Visual Score	
Cytoplasm	0.84 (0.82–0.87)
Nuclei	0.83 (0.80–0.85)

### Outcomes analysis

Select characteristics from the PCa TMA patient cohort are described in Table 3. A total of 508 subjects met eligibility criteria and had suitable tissue available for analysis on the TMA. The mean age at the time of radical prostatectomy was 59.0 years and the median pre-operative diagnostic PSA was 5.9 ng/mL (IQR: 4.6, 9.0). Out of 508 patients, 111 men (21.9 %) experienced PCa recurrence, including 14 men (2.76 %) who died of PCa.

**Table 3:** Characteristics of prostate cancer patients on the tumor tissue microarrays

Variable	Patients (*n* = 508)
Median age (IQR)	59.0 (53.0, 63.0)
Gleason grade	
≤ 6	241
7 (3 + 4)	187
7 (4 + 3)	43
≥ 8	37
Pathologic stage	
Local	344
Regional	164
Median diagnostic PSA (ng/mL; IQR)	5.9 (4.6, 9.0)
Recurrence status	
No	300
Yes	111
Vital status	
Alive	417
Prostate cancer-specific death	14
Other cause of death	71

**Fig. 4:**
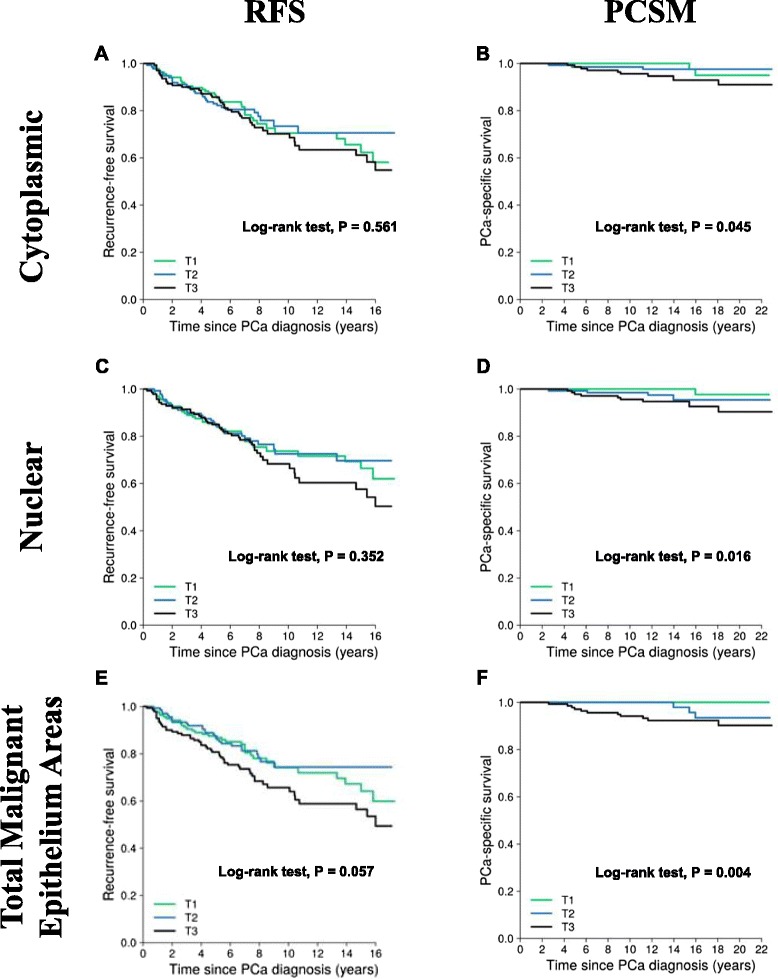
Probability of PCa RFS and PCSM for ERβ2 staining quantified by image analysis. Kaplan-Meier plot for PCa recurrence-free survival using tertiles of ERβ2 intensity quantified by the Cytoplasm algorithm (Aperio) confined to tumor cytoplasm (**a**), tumor nuclei (**c**), or by the Color Deconvolution algorithm (Aperio) for area-based quantification confined to tumor cells including cytoplasm and nuclear staining (**e**). Kaplan-Meier plot for PCa-specific survival using tertiles of ERβ2 intensity quantified by the Cytoplasm algorithm (Aperio) confined to tumor cytoplasm (**b**), tumor nuclei (**d**), or by the Color Deconvolution algorithm (Aperio) for area-based quantification confined to tumor cells including cytoplasm and nuclear staining (**f**)

Kaplan-Meier analysis demonstrated that ERβ2 quantified by total malignant epithelial area image analysis was borderline associated with time to recurrence in univariate analysis (*p* = 0.057; Table 4 and Fig. 4). ERβ2 quantified separately by cytoplasmic image analysis and nuclear image analysis were not significantly associated with time to recurrence in univariate or multivariate analysis (adjusted for clinicopathologic features including age at diagnosis, Gleason score, pathologic stage, and diagnostic PSA level; Table 4 and Fig. 4).

**Table 4:** Hazard ratios (HRs) of PCa recurrent free survival and PCa-specific mortality after radical prostatectomy by ERβ2 staining in tumor epithelium quantified by image analysis (per tertile increment)

	RFS		PCSM	
	HR (95 % CI)	*p*-value	HR (95 % CI)	*p*-value
Cytoplasmic Digital IHC (CytoOD*%PosCyto)				
Univariate	1.07 (0.85, 1.34)	0.561	2.16 (1.02, 4.57)	0.045
Multivariate ^a^	1.06 (0.84, 1.33)	0.624	1.98 (0.93, 4.21)	0.075
Nuclear Digital IHC (NucOD*%PosNuc)				
Univariate	1.11 (0.89, 1.40)	0.352	2.67 (1.20, 5.96)	0.016
Multivariate ^a^	1.00 (0.79, 1.27)	0.999	2.32 (0.99, 5.41)	0.052
Total Malignant Epithelial Area Digital IHC (OD*%Pos)				
Univariate	1.25 (0.99, 1.57)	0.057	5.10 (1.70, 15.34)	0.004
Multivariate ^a^	1.19 (0.94, 1.51)	0.150	4.08 (1.37, 12.15)	0.012

^a^ Adjusted for age at diagnosis (years), Gleason score (≤6, 7[3 + 4], 7[4 + 3], and ≥8), pathological stage (local: pT2, N0/NX, M0; regional: pT3/pT4 or N1-3, M0), and diagnostic PSA level (1 unit increase)

In the univariate analysis of survival, ERβ2 staining was significantly associated with increased risk of PCSM when quantified by cytoplasmic image analysis (HR for each tertile increase = 2.16, 95 % CI 1.02–4.57, *p* = 0.045), nuclear image analysis (HR 2.67, 95 % CI 1.20–5.96, *p* = 0.016), and total malignant epithelial area analysis (HR 5.10, 95 % CI 1.70–15.34, *p* = 0.004). After adjusting for age at diagnosis, Gleason score, pathologic stage, and diagnostic PSA level, ERβ2 staining was significantly associated with increased risk of PCSM when quantified by total malignant epithelial area image analysis (HR 4.08, 95 % CI 1.37–12.15, *p* = 0.012). We did observe a borderline significant association with increased risk of PCSM when ERβ2 staining was quantified by nuclear image analysis (HR 2.32, 95 % CI 0.99–5.41, *p* = 0.052).

## Discussion

The long-term aims of our studies are to examine the association of PCa biomarkers with outcomes at the patient level. Traditionally, we and others in our field have used pathologist visual scoring, which has been shown to have good to excellent intra- and inter-pathologist reproducibility [4, 6, 35]. However, approximations of positive-staining area has only poor to good pathologist reproducibility [6], highlighting the need for improved methods. Although we have demonstrated that the digital image analysis process takes similar quantities of time to complete compared to visual scoring for the current analysis, digital image analysis can be completed by a technician supervised by a pathologist, whereas visual scoring requires significantly more pathologist time. In this study, pathologist time for supervision of the digital image analysis required approximately 10-fold less pathologist time versus visual scoring of the TMAs, similar to our previously published findings [11]. Pathologist availability and efficiency limits visual scoring approaches, especially for large studies [36, 37]. Although we have not evaluated inter-observer pathologist reproducibility in this study, this is another important source of error and thus inter-pathologist and inter-scientist reproducibility studies warrant further investigation.

An initial finding in this study was that the correlation between patient replicate TMA spots was higher for digital IHC measures (in both tumor cytoplasmic and nuclear areas) compared to pathologist scores. These data held up across two independent Analysis Runs and raise an interesting discussion point for tumor heterogeneity and its relation to outcome. High correlation between patient replicate TMA spots in our study conforms to prior research indicating that a relatively small number of cores adequately represent the tumor, although this is highly dependent on the antigen being evaluated [38]. In PCa specifically, a 12 biomarker signature has demonstrated high correlation between patient replicate TMA spots of varying tumor grades indicating that expression of the signature in either high or low Gleason grade similarly informed outcome [39, 40]. Other tumor types may have a higher degree of molecular heterogeneity, and this may contribute to outcome. For example, melanoma tumor cells distant from vasculature demonstrate altered expression of numerous hypoxia-related genes, and potentially react to these localized environments in ways that may be critical for disease aggressiveness [41]. Similar experiments in breast and rectal cancer demonstrate distinct expression patterns at the leading/invasive edges of tumor compared with trailing/center portions, a phenomenon referred to as a “prairie fire” antigen distribution [42, 43]. These data demonstrate that genetic and histopathologic spatial heterogeneity may be reflected in the biologic behavior of cells within distinct tumor areas. Additional studies directly exploring the relationship between heterogeneity of tumor biomarker expression and outcome are needed.

One previous study in PCa identifies a high correlation between digital analysis and ordinal pathologist scores of ERG, SLC45A3, and TMPRSS2 IHC [25]. However, continuous data allows the use of statistical methods more suitable to identifying IHC cut-points of biomarkers with prognostic relevance [2, 7–9]. For this reason, pathology studies have developed semi-quantitative scoring methods [26, 27]. However, multiplying ordinal by continuous data does not produce a true continuous variable but rather a quasi-continuous variable. Problems with quasi-continuous scoring systems are exemplified by Rimm et al. who showed bimodal distribution of pathologist visual scoring due to over-calling of very weak staining as “negative” rather than recognizing the weaker staining which often displays the most variability when quantitatively evaluated [2]. Ideally, much research would move in the direction of truly quantitative methods where staining intensity (perhaps detected using fluorescence methods that have wider dynamic ranges than IHC assays) is reflected on a standard curve of controls with biochemically known target antigen quantity [44].

Schade et al. previously demonstrated that pathologist visual scoring of separate nuclear (intense only) and cytoplasmic (intense only) ERβ2 immunohistochemical staining was associated with a higher risk of PCSM in the same PCa cohort evaluated in the current study [30]. Here, we extended this work by assessing the association between ERβ2 quantified by multiple image analysis methods with PCa outcomes, and identified a significant association with PCSM when ERβ2 was quantified by total malignant epithelial area, and identified a borderline significant association with PCSM when ERβ2 was quantified by nuclear-only staining after adjusting for multiple clinicopathologic factors similar to our previous report [30]. While we observed a significant association with PCSM when ERβ2 was quantified by cytoplasmic image analysis in univariate analysis, this result did not remain significant after adjusting for clinicopathologic factors.

Our current findings build upon the work by Schade et al. showing that ERβ2 is associated with adverse outcomes. However, it is unclear why we found that data obtained by pathologist visual scoring, versus data obtained from the same slides using digital image analysis, yielded slightly different associations with patient outcome metrics (RFS and PCSM). It is possible that low level staining that is present and quantifiable by digital methods, may be interpreted as “negative” by a pathologist relying on visual interpretation of staining intensity [44], resulting in misclassification. Relatively little is published in this area, with some groups suggesting that digital data result in higher associations than visual scoring with outcome metrics [10, 45] and one instance describing a lower association with digital data [46]. Further work, out of scope for the present study, is required to identify factors that may underlie differences of visual versus digital image analysis data and their correlation with outcome metrics.

## Conclusions

Our study, to our knowledge, is the first to assess both reproducibility of pathology visual data and reproducibility of digital methods in the same pathology data set. We demonstrated that digital methods are extremely reproducible across two Analysis Runs which involved re-annotation of tissues, retraining of a pattern recognition algorithm to identify tumor epithelium, and reevaluation and compilation of data. We conclude that computer-aided methods may produce improved datasets and lead to higher quality and more reproducible research, especially in studies involving large sample sizes.

## Abbreviations

CI, confidence interval; ERβ2, estrogen receptor-β2; H&E, hematoxylin and eosin; HR, hazard ratio; IHC, immunohistochemistry; OD, optical density; PCa, prostate cancer; PCSM, prostate cancer-specific mortality; PSA, prostate-specific antigen; RFS, recurrence-free survival; SEER, surveillance, epidemiology, and end results; TMA, tissue microarray

## Additional file

Additional file 1: Raw data for pathologist visual scores. (XLSX 120 kb)
